# Between the Rhetoric of Employability and the Reality of Youth (Under)Employment: NEET Policy Rhetoric in the UK and Scotland

**DOI:** 10.1007/s43151-021-00045-5

**Published:** 2021-06-23

**Authors:** Charlotte McPherson

**Affiliations:** King’s College London, London, England

**Keywords:** NEET, Young people, Policy analysis, Employability

## Abstract

In the UK and Scotland, considerable resources have been devoted to tackling the persistent issue of young people who are, or are at risk of becoming, not in education, employment or training (NEET), a pathologized status that incurs significant penalties for young people and the economy. Using critical discourse analysis, this paper analyses and evaluates policy rhetoric to explore how the NEET ‘problem’, agenda and population are constituted by the UK and Scottish governments. In doing so, numerous unifying and problematic NEET policy tropes are identified, challenging the popular notion of significant policy divergence between the punitive reputation of Westminster and the image of Scottish governance as more socially democratic. Moreover, this paper differs from traditional policy analysis by also evaluating policy from the perspective of young people, drawing on empirical data from a qualitative study of the school-to-work transitions of NEET and marginally employed young people in Scotland.

## Introduction

Tackling the NEET ‘problem’ has been a consistent policy priority in the UK, where it incurs significant costs to the economy and poses both immediate and enduring penalties to young people in terms of diminished life chances, poor long-term labour market prospects and compromised health and wellbeing (Bell and Blanchflower 2011). At the latest count, 11.6% of young people aged 16–24 are currently classified as NEET in the UK Office for National Statistics (2021a), while 8.6% of young people aged 16–19 are categorised as NEET in Scotland according to the most recent figures (Scottish Government 2020a). However, these figures are unlikely to capture the true extent of NEET, both because of the phenomenon of ‘hidden NEETs’ (Jones et al. 2018), and because of the ongoing coronavirus pandemic, which has not only triggered an economic recession but made it logistically challenging for reliable employment statistics to be collected Office for National Statistics (2020).

Like the UK, the devolved Scottish government has prioritised bolstering the engagement of young people in education, employment and training (EET) (e.g. Scottish Executive 2006; Scottish Government 2014a, 2020b). However, Scotland is a particularly interesting policy context, given the Scottish government’s more explicit commitments to delivering social justice to its citizens, a commitment interdependent with the vision of Scotland as a country of distinctively egalitarian values (Leith and Soule 2011). Ideological tensions between the two nations often give rise to the perception of significant policy and political divergence between administrations, an assumption that has been previously challenged in the literature (Tijmstra 2009; Mooney and Scott 2012).

Through critical discourse analysis (CDA), this paper identifies key policy tropes within the NEET policy frames of the UK and Scotland and considers their implications for economically marginalised young people. As this paper goes on to argue, despite appearances—and some instances—of increasing political and policy divergence between the UK and Scottish administrations, in the case of NEET policy, there remains significant overlap in policy positions.

Moreover, while there has been extensive analysis of Scottish and UK policymaking around the issue of NEET and youth unemployment (e.g. Spohrer 2011; Mackie and Tett 2013), and some research considering the policies of both governments (e.g. Adams 2012), this paper goes further by also evaluating policy from the perspective of the young people it purports to serve. Drawing from a qualitative study of the school-to-work transitions of economically marginalised young people living in two areas of Scotland, this paper considers how the Scottish government’s approach to framing and tackling NEET is interpreted and experienced by young people.

Before examining the policies in question, this paper will first place ‘NEET’ terminology in historical and critical context.

## ‘NEET’ Terminology and Usage in the UK and Scotland

In the last few decades, as more young people have engaged in education and training, and the labour market has shifted under the weight of new technologies, policy reform, globalisation and mass deindustrialisation, there has been growing policy interest in young people’s transitions between education and work (Yates and Payne 2006). There has been particular policy emphasis on issues of social and economic exclusion (Conrad 2005) and mounting concern that many young people pose a short- and long-term threat to the economic sustenance and international competitiveness of nation states (Finlay et al. 2010; Keep and Mayhew 2010). This has translated into the phenomenon of categorising, tracking and pathologizing young people who are marginalised from education and work, and who are at risk of derailing from the idealised transition from youth (education) to adulthood (employment) (Furlong et al. 2017).

In the UK, after official recognition of youth unemployment was withdrawn in 1988 following a restructuring of welfare benefits and young people’s reduced entitlements to them, government ministers and researchers began adopting new ways of identifying young people’s labour market positioning and vulnerability (Furlong 2006). In 1999, the NEET label was visibly streamlined into policy rhetoric around young people in the UK in the form of the *Bridging the Gap* report by the newly formed Social Exclusion Unit (Wrigley 2019). This report set the tone for how the NEET status and group of young people it purported to categorise would be understood in the following two decades in the UK, namely, as a problem rooted in individual deficiencies among low-income young people and their families (MacDonald 2011; Wrigley 2019).

NEET terminology has been widely critiqued for two key reasons. Firstly, scholars have criticised its promotion of an inherently deficit understanding of young people that is entangled with classist, often racialised, assumptions and stereotypes around intergenerational welfare dependency and cultures of ‘worklessness’, anchored in a moral underclass discourse that serves to reframe structural problems like mass youth unemployment as products of individual pathology (Levitas 2005; MacDonald 2011). Indeed, NEET terminology has previously not been well received in Scotland, when, in 2007, Fiona Hyslop, then Cabinet Secretary for Education, rejected the term NEET, arguing that it ‘labels unemployed youngsters’, and instead referred to young people outside of education, employment or training as in need of ‘more choices, more chances’, which later became the name of Scotland’s NEET policy strategy (Finlay et al. 2010).

Secondly, NEET terminology has been critiqued for definitional problems related to applying one label to capture a large and diverse group of young people (Yates and Payne 2006; Nudzor 2010). Within the UK and Scotland, the status of NEET applies to a wide range of young people and circumstances, including those actively seeking employment as well as those who are not because of health problems, caring responsibilities or a range of other reasons that are generally not differentiated between in NEET policy and rhetoric, where being NEET is typically assumed to be a problematic state associated with social exclusion and disadvantage (Finlay et al. 2010; Sissons and Jones 2012). Despite these issues, NEET remains the dominant operational term for describing young people outside of EET in the UK, Scotland and many other nations around the world.

## Method

In order to identify key tropes of NEET policy rhetoric in the UK and Scotland, a range of policy documents relating to young people and NEET were analysed using CDA. CDA is a sociolinguistic approach which regards language as social practice and is thus particularly attendant to the relationship between language and power (Fairclough and Wodak 1997; Wodak 2005). This is especially important in the political context, where rhetoric is used to both frame issues in a certain way and to persuade the public of this framing:

Through framing, political elites attempt to define what a public policy issue is about… Through rhetoric, political elites attempt to draw attention to particular features of a policy proposal – while drawing attention away from others. (Koch 1998: 211)

As an analytical tool, CDA has been lauded for its capacity to ‘enrich the conceptualisation—and thus analysis—of policy’ by investigating the role of language in the constitution, contestation and transformation of social problems (Mulderrig et al. 2019: 1). These are particularly valuable assets to one of the objectives of this paper, which is to consider how the NEET ‘problem’, agenda and population are articulated in the policy rhetoric of Scotland and the UK, and the implications of this for young people.

A range of policy documents relevant to young people were analysed. In the UK context, these documents include:

- *Building Engagement, Building Futures* (HM Government 2011a)
- *Positive for Youth* (HM Government 2011b)
- *Post-16 Skills Plan* (Department for Business, Innovation and Skills [BIS] and Department for Education Department for Business, Innovation, and Skills and Department for Education (2016a)
- *Technical Education Reform: The Case for Change* Department for Business, Innovation, and Skills and Department for Education (2016b)
- *Skills for Jobs: Lifelong Learning for Opportunity and Growth* Department for Education (2021)

Policy documents reviewed in the Scottish context include:

- *More Choices, More Chances: A Strategy to Reduce the Proportion of Young People Not in Education, Employment or Training* (Scottish Executive 2006)
- *Skills for Scotland: Accelerating the Recovery and Increasing Sustainable Economic Growth* (Scottish Government 2010)
- *The Government Economic Strategy* (Scottish Government 2011) and *Scotland’s Economic Strategy* (Scottish Government 2015a)
- *Opportunities for All* (Scottish Government 2012)
- *Developing the Young Workforce* (Scottish Government 2014a, 2018, 2019)
- *Education Working for All!* (Scottish Government 2014b)
- *Consequences, Risk Factors and Geography of Young People Not in Education, Employment or Training – Research Findings* (Scottish Government 2015b)
- *Young Person Guarantee: No-One Left Behind* (Scottish Government 2020b)

In the case of the UK context, the lack of more recent documents under review is due to the lack of substantial updates to the UK’s youth unemployment strategy that was implemented in 2011 (HM Government 2011a). In the years since, the UK government has instead focused on extensive reforms to technical education, which is perceived to be pivotal in addressing youth unemployment, among other challenges (Department for Business, Innovation, and Skills and Department for Education 2016a, b; Department for Education 2021). The Scottish government has taken a more intensive approach to tackling NEET, introducing a youth employment strategy in 2014 that has been consistently updated and linked into its recent Covid-19 response around young people (Scottish Government 2020b).

The author carefully reviewed each policy document, looking for dominant trends in how the NEET ‘problem’, agenda and population were represented, and how these portrayals differed or overlapped with one another. Trends were identified from a close reading and re-reading of each document and were then mapped thematically. Finally, the themes identified in the Scottish policy documents were considered against the empirical data from the project described below.

In addition to CDA, this paper draws on empirical data from the author’s qualitative doctoral study of youth transitions and social justice conducted in Scotland between 2016 and 2019 (see McPherson 2019). Some of the policy analysis presented in this paper is based on analysis conducted for this study, where UK and Scottish policymaking was analysed to contextualise the school-to-work transition experiences of 42 economically marginalised young people (aged 14–29) from two contrasting areas of Scotland, an urban city and a small semi-rural town.

Using Nancy Fraser’s (1998) theory of social justice, this study explored whether young people felt they were experiencing social justice during their transitions in Scotland and how they navigated these transitions. While only 7 of the 42 young people were classified as NEET at the time of fieldwork, all over the age of 16 had experience of being NEET, were marginally employed and/or regularly churned between unemployment and underemployment. During interviews or focus groups, participants frequently referred either directly or indirectly to policy approaches and rhetoric around youth unemployment, and this data is presented throughout the paper alongside the policy analysis, providing insight into how policy narratives square with young people’s experiences.

## Key Tropes of NEET Policy in the UK and Scotland

This paper now moves to a discussion of several defining tropes that feature prominently both north and south of the border. Excerpts of testimony from young people that speak to and/or challenge this rhetoric are included throughout. Young people’s names have been protected with pseudonyms.

### Young People as (Risky) Economic Subjects

Young people are overwhelmingly positioned as economic subjects in the UK and Scottish policy discourses. This characterisation has been widely critiqued by scholars (e.g. Kelly 2006; Wyn 2009), who have argued that the resulting policy fixation on young people’s movements between EET denotes youth as a time of ‘transition’ and reductively conflates adulthood with obtaining employment (Cohen and Ainley 2000).

The construction of youth as transition, and the corresponding policy fixation on young people moving smoothly and linearly between education and work, has generated a dichotomy between ‘on track’ and ‘at risk’ youth, based on young people’s ability to achieve normative yardsticks of adulthood (i.e. getting a university degree, gaining a full-time job) (Cuervo and Wyn 2014). Such sentiments are widely evident in UK policy rhetoric, which draws on neoliberal notions of individual responsibility to encourage young people to ‘be the authors of their own story’ (HM Government 2011b) and become ‘ideal citizen-workers’ (Pimlott-Wilson 2017: 289).

The binary between ‘on track’ and ‘at risk’ youth valorises seamless transitions between education and employment, while pathologizing those young people whose transitions are more protracted, fragmented and precarious (Cuervo and Wyn 2014). Policy labels like NEET, which define young people by what they are not (Nudzor 2010), flow from this framing of young people as (risky) economic subjects, where the implication is that NEET status is the result of individual failures and deficiencies.

As in the UK policy context, young people in Scotland are primarily understood as economic subjects through whom the future of the economy can be either assured or threatened:

Learning, living and working in today’s economy requires young people to be flexible, adaptable and to have the on-going capacity to develop knowledge and skills. This investment in our young people is essential for the future growth of our economy. (Scottish Government 2011: 61)

Despite some resistance in Scotland to how the NEET label can stigmatise and blame young people, it nonetheless proliferates Scottish policy documents, though is often couched in softer rhetoric that emphasises structural barriers and responsibilities rather than individual ones (e.g. Scottish Government 2019). While there is less of an emphasis on denigrating young people for being NEET in Scottish policy, the corresponding dichotomy drawn between ‘on track’ and ‘at risk’ youth/transitions that flows from defining young people economically is equally pervasive in Scottish NEET policy, where there is a strong emphasis on ‘at risk’ youth:

Out of school hours activities can be effectively targeted to help re-engage and motivate young people at risk of becoming NEET. (Scottish Executive 2006: 21)

While the rhetoric may be softer in Scottish policy, participants in the qualitative study were often sensitive to their positioning as ‘risky’. One participant described his guidance teacher explicitly referencing a divide between ‘at risk’ young people with those felt to be more ‘on track’:

I remember him kicking off a class about careers at the start of what would have been my fourth year… He said he’d just be honest and that there are some of us who are more on track and they aren’t really worried about, like, they’ll go off to uni or do whatever, but that this group, we were seen as more ‘at risk’ so they were more worried about where we were going to end up after school… It made me and my friends feel shit to be honest, being singled out and labelled like that. (Casey, 25)

Critically, moreover, Casey felt he and his other ‘at risk’ peers had not been provided with valuable additional support with their transitions, but instead lessons had been focused on ‘improving’ their work ethic and attitude. Just as in the UK context, this contemporary fixation on marginalised young people as ‘risks’ means that Scottish policy approaches are often based on calculations of risk on the basis of individual characteristics rather than on structural barriers and inequalities (Mackie and Tett 2013).

### Employability and Personal Responsibility

Themes of individual deficiency have become increasingly dominant in NEET policy in the UK. This has most clearly manifested through notions of ‘employability’, a form of supply-side orthodoxy that reframes youth unemployment as an individual rather than structural problem (Crisp and Powell 2017). Supply-side orthodoxy is typically expressed in one or both of two ways. Firstly, supply-side orthodoxy is implied through classed concerns about the ‘character’, work ethic and attitudes of economically marginalised young people and their families, with references to alleged intergenerational cultures of worklessness and welfare dependency (e.g. Hancock 2015).

This was exemplified when Matt Hancock, then Paymaster General, buttressed the announcement of a ‘no excuses’ youth workfare programme with classist stereotypes about economically marginalised young people and their families, where youth unemployment was positioned as a product of laziness and cultures of worklessness:

We are determined to fulfil our commitments to end the welfare culture that is embedded in some of Britain’s most vulnerable communities. By working across government to make sure that every young person is in work or training… and making sure that a life on benefits is simply not an option, we want to end rolling welfare dependency for good, so welfare dependency is no longer passed down the generations. (Hancock 2015)

The second way in which supply-side orthodoxy emerges in policy rhetoric is through narratives of skills shortages, which have become more prevalent in the UK and Scotland since the New Labour government of 1997–2010 (Mok and Neubauer 2016):

We face a major challenge: the pressing need for more highly skilled people, trained effectively, to grow the economy and raise productivity… We need young people and adults to have the skills and knowledge that better equip them for employment in the 21^st^ century, in order to meet the demands of the future. (Department for Business, Innovation, and Skills and Department for Education 2016a: 10)

The articulation of NEET as primarily a problem of a lack of *employability* among young people rather than of opportunities echoes in Scotland. Where this has often been articulated through scrutiny of the presumed attitudes and work ethic of young people in the UK, this rhetoric has tapered off in Scotland, where there is a greater emphasis on the state extending opportunities to young people and dismantling barriers to inclusion (e.g. Scottish Government 2012, 2014a). However, employability remains a consistent and increasingly foregrounded feature of Scottish policy:

The Review has highlighted the need for additional focus on employability… This is central to our approach to education as we continue efforts to develop the workforce the economy requires. (Scottish Government 2018: 14)

Many of the participants were cognisant of the employability policy agenda in Scotland and were broadly critical of this approach and the assumptions they felt underpinned it. Damian, 23, felt that the emphasis on employability served to blame young people for their own labour market disadvantage:

I think that whole ‘be employable’, ‘get out there and get employable’ stuff is what annoyed me the most about being unemployed before because it makes out there’s something wrong with you almost, and not the system... It ignores the fact that there are no decent jobs out there for young people, who actually try really hard to get skills and qualifications and to move up in the world but can’t, not because we are lazy but because the doors feel closed to us.

The Scottish government has increasingly legitimised the idea that a skills crisis among young people is the leading cause of youth unemployment (Valiente et al. 2020). As in the UK, policies of lifelong learning with an emphasis on VET routes have abounded from this position, perceived to be vital to addressing the twin priorities of reducing the NEET population and securing a pipeline of adequately skilled, competitively qualified future workers for the labour market (Scottish Government 2014b, 2018).

In both the contemporary UK and Scotland, then, youth unemployment is traced to young people not possessing adequate skills to meet the demands of the market; youth unemployment is still framed as the product of individual deficiency, but is less about character flaws than perceived skills deficits. The ‘skills crisis’ policy position, and notions of supply-side orthodoxy more broadly, has been critiqued for implying that young people are to blame for their own disadvantage (Crisp and Powell 2017), not least because the current youth cohort is the highest educated in history (Education and Employers 2015).

Most of the working-age participants in the sample possessed a range of skills, educational qualifications and/or work experience. Participants who had engaged with further education after school were broadly critical of the framing of young people’s skills deficits as a leading factor in their labour market disadvantage, using their parents’ typically lower levels of skills and qualifications, but more streamlined transitions into employment, as a point of reference. Damian, 23, was one of only two participants in a full-time job aligned with their skills and qualifications. He described it being a long and difficult process, however:

I’m obviously chuffed to bits with the job I have now, but it was incredibly difficult to get there, and in ways that I don’t think the previous generation necessarily appreciates or had to deal with themselves… You know, my parents, who have no education whatsoever, were in more secure employment than I could access with a degree for such a long time, just as one example.

Many of the young people had done as the UK and Scottish policymaking encourages and invested in further education and training in order to enhance their ‘employability’, but most had not experienced any corresponding advantages in the labour market. Nicholas, 23, had studied various electrical engineering courses at college but had ‘hit a wall’, forcing him back into the labour market where he had struggled to find any jobs matched to his skillset:

It really, really angers me that you are told to go to school, do well in school, go specialise in something at college and then you will get rewarded… So, you go do that, I went to college for years actually, yet here I am, still doing the same shitty jobs despite being qualified to work in engineering.

Participants felt strongly that their goals for the future were not only aspirations but expectations, justified by their hard work and their level of skills and experience, which often significantly exceeded the skills and qualifications of their parents. Instead of being able to identify or work in more skilled, higher paying jobs, however, they felt trapped in low-skilled, poorly paid jobs that did not align with their skills, qualifications or aspirations; something rarely acknowledged in policy.

### The Idealisation of Employment

A consistent feature of NEET policy in the UK and Scotland has been the argument that getting a job is not only the best solution to a NEET status, but also carries a range of other benefits, whether through addressing young people’s alleged character deficits or cementing young people as adult contributors to society:

Part-time jobs can help young people understand the adult world of work, develop new skills, and learn the habits of punctuality and persistence. (HM Government 2011b: 37)

Employment is also often presented as an opportunity that is widely available to young people (e.g. HM Government 2011a, b; Scottish Government 2014a, 2018), something young people need only reach out and grab:

Ultimately, it is about the future workforce, our young people, making informed and ambitious choices about jobs and careers, ready to take their place in the world as effective contributors. (Scottish Government 2014a: i)

In either presentation, work is generally depicted as an uncomplicated and universally positive opportunity for young people, with policy interventions overwhelmingly focused on bolstering employability and securing youth employment. One example of the perceived importance of work is the contemporary policy fixation on making education more explicitly industry-facing (Scottish Government 2014a, b; Department for Business, Innovation, and Skills and Department for Education 2016a, b). This delineation of learning to maximise employability is evident in Scotland’s education strategy, which proposes that employability training should begin as early as primary school:

A shift is clearly underway from purely the provision of learning to more focus on employability and skills required to meet market demand… This trend must continue… There should be a continuum from primary school right through into employment. (Scottish Government 2014b: 4)

These sentiments are mirrored in the UK government’s *Post-16 Skills Plan* (Department for Business, Innovation, and Skills and Department for Education 2016a, b: 12), a substantial reform to education which envisages giving employers the lead in designing curricula and setting qualifications:

Employers, large and small, will sit at the heart of a dynamic skills system to ensure the day-to-day training and education that individuals receive genuinely meet the needs of industry.

The overriding emphasis on employment (and unemployment) driving this reframing of education is also problematic because it obscures the more complex reality of (under)employment in the contemporary context. This reflects in the labour market experiences of the participants. While 9 were NEET or long-term unemployed, the overwhelming majority of working-age participants were less affected by the quantity of job vacancies available than by the quality of these opportunities. Michael, a 23-year-old classified as NEET, described the challenges of finding a job with which he could make ends meet:

I sit there every day, right, no word of a life, and I’m scrolling and scrolling and there is absolutely nowt. Nothing. Well, there is, there’s the usual shite, cleaning and shop work, factory stuff sometimes, but it’s zero hours, minimum wage, part-time and I can’t live on it, it’s not possible.

All but 5 of the 19 working-age participants in employment were underemployed on part-time, zero hours or otherwise fixed-term contracts in hospitality, services and retail. Numerous participants described the challenges of making ends meet on these jobs, sometimes juggling multiple jobs in order to try and accumulate a full-time wage. Imogen, 27, worked a zero hours job in social care and found her lack of financial security a constant source of stress:

It’s a stress every single week in terms of what shifts will they give me, will I have enough for the bills and to put to the debt, and then my partner is employed zero hours as well… You have no control, it’s just not a way to live… Constantly worrying about meals and bills and heating and rent and living day to day.

The problem of low-quality employment is also consistently reflected in labour market statistics. Underemployment has historically not been tracked to the same extent and reliability as employment in the UK, with healthy employment rates often obscuring the problem of underemployment. The data that is available suggests that underemployment is a growing problem in the UK. At the latest count, the underemployment rate for the UK stood at 8.9% for 2020, up from 7.5% in 2018 Office for National Statistics (2021b). Figures produced by Statista (2020) illustrate that the number of people employed on zero hours contracts reached an all-time high of over 1.05 million in 2020. Young people aged 16–24 are the most likely to be in zero hours employment, with 9.9% of this age group on these contracts, compared with just 1.7% of those aged between 35 and 49 (ibid.).

The destabilisation of the labour market has significantly eroded job security and normalised insecure forms of working, particularly among young people, who are the most likely to be involuntarily employed on zero hours or otherwise fixed-term contracts (Lewchuk, 2017). As a result, many of the jobs available to young people now do not protect against poverty in ways that working-class jobs previously did, something that is poorly accounted for in both policy frames that speak of the virtues of employment and the need for young people to upskill (Roberts 2012; Crisp and Powell 2017). Taken together, this suggests that there is a significant gap between the policy idealisation of employment as a widely available and unproblematic opportunity for young people, with the more complex reality of in-work poverty.

Moreover, these issues are likely to be significantly compounded by the economic impact of the ongoing coronavirus pandemic, which has significantly reduced the quantity of job opportunities, and particularly those lower quality, entry-level jobs that overwhelmingly employ young people (Institute for Fiscal Studies 2020). In the last quarter (December 2020–February 2021), youth unemployment has increased by 10%, the number of 18–24s claiming unemployment-related benefits has increased by 110% and the number of job vacancies fell by 22.7% (House of Commons Library 2021; Office for National Statistics 2021b).

### Equality of Opportunity

Finally, a consistent feature of NEET policy discourse has been a commitment to equality of opportunity. Indeed, despite the emphasis on personal responsibility and employability, policy documents are replete with commitments to providing young people with resources and opportunities. Premised on a meritocratic vision of British society, this rhetoric of equality of opportunity is nonetheless typically superimposed over neoliberal terminologies of responsibility, where the state’s role is clarified but the obligations, and perceived deficits, of young people and/or their families are ultimately underlined:

It is clear that we need to do more to help many young people who are at risk of dropping out of society to develop a much stronger, clearer sense of responsibility and respect for others, real aspirations and pride for themselves. (HM Government 2011b)

In Scotland, the emphasis on equality of opportunity is significantly stronger, in that it is bound up in the curation of the Scottish government—and Scotland itself—as grounded in ideals and principles of social justice and equality. Scottish NEET policy documents are accordingly saturated with such terminology:

This strategy aims to promote equal access to and participation in skills, career information, advice and guidance and learning activities for everyone. It is intended to promote equality of opportunity to those who face persistent disadvantage and to improve the numbers of people economically active across all groups within society. (Scottish Government 2010: 6)

Critically, however, this policy commitment is to equality of *opportunity,* not outcome (Mackie and Tett 2013), which is problematic given that the mere existence of opportunities is not enough to level the playing field for many young people, whose capacities to access and benefit from opportunities are heavily intertwined with enduring and chronically overlooked socioeconomic inequalities (Hine and Wood 2009).

The young participants all lived in deprived neighbourhoods in two areas of Scotland. They spoke widely about the barriers they felt their social class background presented, referencing the stigmatising attitudes of education practitioners and employers, for example, and of lacking the money, home life or social capital to identify, access or benefit from EET opportunities. Participants also often described experiencing discrimination around their transitions. Jackson, a 15-year-old high school student had expressed an interest in going to university to study business to his guidance teacher, but described being met ‘with a weird look’ and being given ‘all these leaflets about working in the local supermarkets’.

Further barriers identified by participants pertained to age and generation, with many young people feeling excluded from opportunities due to the stigma attached to young people in the labour market, where they can often be perceived as ‘risky’ to hire next to older and more experienced candidates (see CIPD 2013). When assumptions about the work ethic, aptitude and reliability of the participants as young people combined with their socioeconomic status, they often felt they were up against it in the labour market:

Sometimes when I’ve filled in applications and done interviews and that before, it’s just been like, what’s the point? I’m a teenager from a supposedly dodgy area and you know they aren’t going to take a chance on you type thing… It’s hard to know what to do about that. (Lloyd, 19)

Finally, many of the young people commented on feeling a sense of intergenerational injustice about their school-to-work transitions. There has been extensive commentary on the greatly changed transition context for current youth cohorts as compared to that of previous generations (e.g. Blatterer 2010). Young people frequently referred to a disjuncture between their parents’ transitions and experiences of employment with their own, and felt policy did not acknowledge the extra challenges they were facing:

I feel like I have no map to follow because nothing seems certain or, I guess, how it maybe used to be for the other generations… There isn’t a set path or whatever to follow. It’s a really confusing time to be trying to figure out all this adulting stuff… There’s a lot of blame on young people but I feel like people in power don’t get that it’s much more difficult to be young these days. (Denise, 19)

Indeed, while it is now widely recognised in research that young people’s school-to-work transitions are more likely to be protracted, fragmented and unpredictable than the relatively predictable, linear transitions of previous generations (Punch 2015), policy in the UK and Scotland has largely failed to acknowledge these generational shifts, or the barriers and inequalities they can present for young people (Furlong et al. 2011). In both NEET policy frames, it remains unclear how these barriers to accessing and benefiting from an equality of opportunity are to be dismantled (Scott and Mooney 2009).

## Conclusion

Using CDA and drawing from qualitative data collected from 42 economically marginalised young people in Scotland, this paper has identified and problematised several unifying tropes in NEET policy rhetoric in the UK and Scotland. It has argued that despite popular perceptions—and some instances of political and policy divergence—the rhetoric that underpins NEET policy is in fact more similar than it is different between the two neighbouring nations. This congruence is evident across a range of interlinked topes, including the positioning of young people as risky economic subjects; an emphasis on employability and personal responsibility; the idealisation of employment; and a rhetorical commitment to equality of opportunity.

More differences are observable between Scotland and the UK’s policy responses around young people in the midst of the coronavirus pandemic, however. Aside from installing the UK-wide Kickstart scheme, designed to encourage employers to hire young people, the UK government have maintained their focus on extensive reforms to the skills system and technical education (Department for Education 2021). The Scottish government have taken a more intensive approach, identifying young people as a clear priority in their plans for economic recovery from the pandemic. They have introduced a young person’s guarantee (Scottish Government 2020b), which commits to providing every young person between 16 and 24 with a place in work, education or training. Encouragingly, this commitment is to ‘fair employment’, with the goal being to ‘eradicate in-work poverty for 16-24-year-olds’ (p.7). Calls for a similar scheme to be introduced in the UK are yet to be heeded by Westminster, and there are concerns about whether Kickstart will be sufficient to mitigate against the jobs crisis facing young people during and beyond the pandemic (Thomas 2020).

A further argument made in this paper is that Scotland and the UK’s policy frames have been largely ineffective in articulating and tackling the NEET ‘problem’. While the unique impacts of the pandemic have posed significant challenges, the youth unemployment rate has consistently far exceeded the general unemployment rate in both countries and labour market data suggests that youth unemployment was rising in both the UK and Scotland since the summer of 2019 (Scottish Government 2020c; Statista 2021). In 2019 in Scotland, the employment rate for young people stood at 53.2%, 5.9 percentage points lower than in 2018 (Scottish Government 2020c).

Moreover, while youth unemployment rates *have* fallen for sustained periods in recent years in both nations, with Scotland’s youth unemployment strategy delivering its target of reducing youth unemployment by 40% by 2021 in 2017 (Scottish Government 2019), for example, the quality and nature of the jobs overwhelmingly available to young people has been overlooked in policy frames too fixated on the issue of unemployment. As the literature and labour market data has evidenced, however, underemployment is arguably a more pervasive problem facing young people today than unemployment, with extensive evidence pointing to the declining quality of jobs overwhelmingly occupied by young workers in terms of pay, prospects of progression, skill level or security (TUC 2018; Office for National Statistics 2019; Statista 2020). Furthermore, in both countries, concerns were mounting before the pandemic about stubbornly wide attainment gaps among young people at all levels of education (Kirby and Cullinane 2016; McEnaney 2020).

This paper has also problematised Scottish and UK NEET policy approaches by engaging directly with the perspectives and experiences of working-class young people, who are typically marginalised in discussions and constructions of policy (Lister 2007). Incorporating the voices of the young people who are purportedly served by NEET/youth unemployment policy complements the aims of CDA, where the focus is on the power of language in framing policy problems, their causes and their remedies.

Engaging with economically marginalised young people revealed three key insights. Firstly, participants were sensitive to how both the issue of NEET/youth unemployment and they themselves were framed in policy. The construction of young people as ‘risky’ and the framing of NEET as rooted in individual deficits resonated in their experiences of discrimination and labelling in education and the labour market. Secondly, the assumptions underpinning NEET policy were consistently challenged—both directly and indirectly—by the experiences of the participants. They generally possessed a range of skills, qualifications and/or work experience, for example, but were struggling to benefit from these credentials in the labour market. Thirdly, the young people found policy approaches to NEET/youth unemployment to be largely ineffective. Resounding critiques were made of the employability thesis that drives both policy frames, for example, with poor experiences and returns from not only specialist further education but also more generic employability initiatives. Many participants instead pointed to the need to dismantle class-, age- and generation-specific barriers to employment, and the importance of enhancing the quality as well as quantity of EET opportunities.

While policy critiques are familiar when it comes to the UK government, they are far less so in the case of the Scottish government, whose consistent curation of their governance as set against that of Westminster’s has often contributed to a comparative lack of scrutiny of Scottish social policy. Studies that have subject the Scottish policy context to scrutiny have raised important critiques of youth-focused social policy in Scotland, including Mackie and Tett’s (2013) argument that youth policy fails to deliver social justice to young people in Scotland, and McPherson’s (2019, 2020) empirical research with marginalised young people in Scotland coming to the same conclusion.

It is important that more research be conducted that continues to subject NEET policy and its rhetoric to scrutiny, particularly in the Scottish context and ideally engaging with young people themselves. Research of this kind is particularly vital at the time of writing, however, given the unprecedented global COVID-19 pandemic and its disproportionate impact on young people (International Labour Organisation 2020). The sheer urgency of the situation facing young people in the short and long term as a result of this pandemic, which has exacerbated existing inequalities and disadvantages as well as engendering new ones (McPherson 2020), demands that policy in the UK and Scotland must break from tradition in order to extend its commitment to social justice to marginalised young people out of abstracted rhetoric into reality.
